# Reconstruction of Identity and Meaning in the Postpartum Period: Women’s Experiences of Social Vulnerability and Existential Transition—A Phenomenological Study

**DOI:** 10.3390/healthcare14050693

**Published:** 2026-03-09

**Authors:** Aycan Şahin, Fatih Şahin, Leyla Sezgin

**Affiliations:** 1 Department of Nursing, Faculty of Health Sciences, Muş Alparslan University, 49100 Muş, Turkey; fatih.sahin@alparslan.edu.tr; 2 Muş Health Services Vocational School, Muş Alparslan University, 49100 Muş, Turkey; leyla.sezgin@alparslan.edu.tr

**Keywords:** identity reconstruction, social vulnerability, obstetric and gynecological nursing, psychiatric nursing, meaning-making

## Abstract

**Highlights:**

This study reconceptualizes the postpartum period as a multidimensional existential transition. “Rebirthing the self” is proposed as a novel conceptual framework to explain identity reconstruction in vulnerable contexts. Women’s experiences reveal the coexistence of fracture, negotiation, and silent resilience in early motherhood. Findings call for socially and existentially informed postpartum care models.

**What are the main findings?**

- Identity reconstruction in the postpartum period extends beyond role adaptation and reflects a multidimensional existential transition.
- Social vulnerability intensifies the ontological transformation and reshapes meaning-making processes.

**What are the implications of the main findings?**

- Maternal health services should integrate existential and psychosocial dimensions into postpartum care models.
- The proposed concept of “rebirthing the self” contributes a novel framework to maternal and women’s health literature.

**Abstract:**

**Background:** The postpartum period represents a critical transitional phase in which women experience profound changes in identity, meaning, and social roles. This process is often shaped by social vulnerability and existential transformation, yet remains insufficiently explored from a phenomenological perspective. This study aimed to explore how women reconstruct identity and meaning during the postpartum period within the context of social vulnerability and existential transition. **Methods:** This qualitative study em-ployed an descriptive phenomenological approach in accordance with the COREQ guidelines. Data saturation was achieved with 20 mothers of infants aged 0–12 months who were purposively selected from a province in eastern Türkiye. Data were collected through semi-structured face-to-face interviews and analyzed using Colaizzi’s phenomenological method. Credibility was ensured through participant validation, reflexivity, and team-based analysis. **Results:** Four themes emerged. Fracturing of Existence indicated an ontological shift from “I” to “we,” reflecting a metaphorical rebirth of the self. Invisible Burdens revealed that societal expectations and insufficient social support intensify psychosocial vulnerability. Re-Tailoring the Self demonstrated that maternal identity is dynamic and continuously negotiated between the past and emerging self. Construction of Silent Resilience showed that women develop strength alongside vulnerability through internal resources, spirituality, and everyday practices of hope. **Conclusions:** The postpartum period involves a multilayered reconstruction of identity and meaning beyond role adaptation. During this existential transition, women not only give birth to a child but also reconstruct their own existence, metaphorically giving birth to themselves.

## 1. Introduction

Childbirth is not merely a biological event but a transitional process that reshapes a woman’s identity, social roles, and existential framework of meaning. With the advent of motherhood, a woman undergoes a profound transformation both physically and psychologically. As she bids farewell to her previous life experiences, she begins to shape her identity as a “mother.” This process simultaneously entails the dissolution and reconstruction of identity [1]. For women, this process represents not only the learning and internalization of the maternal role but also a simultaneous transformation in which existence itself is reinterpreted and redefined [2].

This transformation brings multidimensional tensions at both the individual and societal levels. Women may fluctuate between traditional motherhood norms and personal autonomy; this duality can create a domain of social vulnerability that threatens identity coherence [3]. Social vulnerability in this context refers to conditions such as limited social support, economic constraints, social expectations surrounding motherhood, and restricted access to supportive resources that may intensify women’s psychosocial burden during the postpartum transition.

Phenomenological work in Sweden shows that new mothers often oscillate between their former selves and emerging maternal identities, with this period shaped by feelings of inadequacy, anxiety, and loneliness [4].

Similarly, qualitative evidence from Iran highlights struggles to “regain balance,” emotional fluctuations, loss of control, and identity confusion in the postpartum period [5], while studies from Spain underscore identity splitting under social pressures and the ideal of “perfect motherhood” [6].

Within this transition, social support and a sense of social belonging function as key determinants of identity construction. A lack of support is associated with diminished self-worth and depressive symptoms [7], indicating that vulnerability in motherhood is simultaneously psychological and social. Research on postpartum depression further emphasizes that when women experience erosion of meaning, identity fragmentation may deepen and evolve into an existential crisis [8].

From an existential perspective, the postpartum period is a process marked by heightened awareness of the meaning of life, mortality, and the depth of relationships [9]. During this period, women establish a new relationship not only with their children but also with their inner selves. This situation may initiate a process of spiritual growth and “rediscovery of the self” for some women, while for others it may signify existential loneliness [10].

Educational attainment, professional life, and social environment are also significant factors in the psychological and identity transformation of new mothers. One study has also emphasized motherhood among professional athletes as a restructuring process that conflicts with their career identity [11]. Recent studies have indicated that Meleis’s “Transition Theory” provides an important framework for understanding this process. Nursing practices grounded in this theory strengthen mothers’ sense of self-integrity, enhance role satisfaction, and facilitate identity integration [12].

Taken together, the literature indicates that reconstruction of identity and meaning in the postpartum period reflects a profound transformation shaped by social support, cultural norms, spiritual beliefs, and personal practices of resilience [13,14,15].

Within this conceptual framework, the concept of “rebirthing the self” denotes a multilayered transformation in which a woman’s sense of self, meaning-making framework, and relational positioning are simultaneously reconstructed during the transition to motherhood. This process extends beyond the mere adoption of a new role and involves a dynamic reconstruction of the self, in which previously established layers of identity are questioned, reinterpreted, and gradually integrated with the emerging maternal identity. From a theoretical standpoint, this transformation aligns with Transition Theory, which defines critical life transitions as processes through which individuals reconstruct their identities and renegotiate their social roles [12]. Indeed, the literature on the formation of maternal identity indicates that motherhood generates a profound identity transformation that reshapes an individual’s sense of self, relational bonds, and life priorities [13,14,15]. However, the postpartum period is also regarded as a distinctive experiential domain in which individuals reconstruct the meaning of life and confront existential questioning [9,10]. In this context, “rebirthing the self” represents an existential process of reconstruction through which a woman not only gives birth to a baby but also reconstitutes her own sense of self within new meanings, responsibilities, and relational contexts. Thus, the concept conceptualizes a distinctive domain of transformation located at the intersection of identity reconstruction, meaning-making processes, and the negotiation of social expectations that arise during the postpartum transition.

Despite the growing body of literature on the transition to motherhood, limited research has explored how identity reconstruction and meaning-making simultaneously unfold within contexts of social vulnerability from a phenomenological perspective; therefore, this study aims to provide an in-depth understanding of women’s identity transformation and existential experiences during the postpartum period by examining the lived meaning of motherhood during this critical life transition.

## 2. Materials and Methods

### 2.1. Study Design

This study was designed as a qualitative research project aimed at gaining an in-depth understanding of women’s identity, meaning, and existential experiences following childbirth. In the conduct and reporting of the study, the COREQ (Consolidated Criteria for Reporting Qualitative Research) checklist, developed to ensure transparency and rigor in qualitative research, was used as a guiding framework. All stages of the research were planned and conducted in accordance with the principles of this guideline. Accordingly, the study aimed to holistically interpret women’s identity transformation in the postpartum period, considering both their individual lives and the broader social context. The reporting of this study followed the COREQ (Consolidated Criteria for Reporting Qualitative Research) checklist to ensure transparency in qualitative reporting.

### 2.2. Study Group

The population of this study consisted of mothers with infants aged up to 12 months who received follow-up and monitoring services at Muş State Hospital and Family Health Centers (FHCs) located within the provincial borders of Muş. The sample of this study consisted of mothers with infants aged 12 months or younger who were purposively selected from the target population and who agreed to share their experiences regarding identity, self-concept, and social roles during the postpartum period. This sampling approach enhanced the credibility of the study and contributed to the validity of the findings.

In this study, social vulnerability was not treated as an a priori label; rather, it was approached multi-dimensionally based on participants’ socioeconomic and social conditions, as well as the lack of support, isolation, and difficulties in access that emerged in their interview narratives.

Inclusion criteria included the following:

- Residing within the provincial borders of Muş;
- Actively receiving services from Muş State Hospital or Family Health Centers (FHCs);
- Having an infant aged 0–12 months;
- Actively experiencing motherhood during the postpartum period;
- Providing voluntary consent to participate in the study.

Due to the nature of phenomenological studies, which prioritize the depth of experience rather than numerical representativeness, the sample size is determined by data saturation, and recruitment is concluded once saturation is achieved [16,17]. As a result of the interviews conducted, data saturation was achieved with 20 participants, and the sample was finalized at this point.

### 2.3. Data Collection Tools

#### 2.3.1. Personal Information Form

This data form was developed by the researchers following a review of the relevant literature. The form was designed to determine the participants’ basic sociodemographic characteristics and included information such as age, educational level, marital status, number of children, employment status, and time elapsed since childbirth. The purpose of this form was to obtain descriptive data necessary to better understand the personal and social context of each participant’s postpartum experience.

#### 2.3.2. Semi-Structured Interview Form

The semi-structured interview form was developed by the researchers in line with the relevant literature to examine in depth the psychosocial, identity-related, and existential experiences of women during the postpartum period. To ensure content and face validity, the form was refined in accordance with the opinions and recommendations of three expert academics experienced in qualitative research and in the fields of women’s health, mental health, and public health. The interview form consisted of nine open-ended main questions. Prior to the main data collection, the interview guide was pilot tested with two mothers to assess the clarity and comprehensibility of the questions; these pilot interviews were not included in the final analysis. The questions were designed to elicit detailed accounts of participants’ experiences related to postpartum existential transition (identity and search for meaning), social vulnerability and support experiences, as well as identity reconstruction and personal transformation [18,19,20]. The questions included in the semi-structured interview form were as follows:

1. How would you describe your experience regarding the meaning of life after childbirth?
2. Could you describe how you define yourself after becoming a mother?
3. How did you experience the postpartum period in your life?
4. How would you evaluate the support you received from your social environment after childbirth?
5. How did you perceive societal expectations of motherhood in relation to your own experience?
6. How did you experience your position within social relationships during the postpartum period?
7. How would you explain the impact of the experience of becoming a mother on your sense of identity?
8. How would you describe the relationship between your pre-motherhood self and your postpartum self?
9. How do you relate your maternal role to the other roles in your life?

### 2.4. Data Collection Process

This study was conducted throughout January 2026 in Muş, Türkiye. Participants were selected on a voluntary basis and were initially contacted at the obstetrics and gynecology outpatient clinic where the data were collected. They were provided with detailed information regarding the purpose, scope, procedures, and ethical principles of the study prior to inclusion in the interviews. Written informed consent was obtained from all participants, and the necessary assurances regarding confidentiality and data protection were provided. Two eligible individuals declined to participate.

The interviews were conducted face to face in a private consultation room at the obstetrics and gynecology outpatient clinic to ensure privacy and safety. No other individuals were present during the interviews. With the participants’ consent, the interviews were audio-recorded. Prior to the main interviews, a brief preliminary meeting lasting approximately 10–15 min was held to establish rapport and facilitate the participant’s adaptation to the interview process. The content of this preliminary session was not included in the research analysis. Each main interview lasted between 30 and 60 min, depending on the participant.

The data were used solely for scientific research purposes; all recordings and field notes were stored in accordance with ethical standards, ensuring confidentiality and data security. All interviews were conducted by the first author, while data analysis, theme development, and interpretation of the findings were carried out collaboratively by the research team through joint evaluation and mutual discussion. Throughout the process, principles of therapeutic communication were carefully observed, participants’ psychological comfort was prioritized, and they were informed of their right to withdraw from the interview at any time, either during or after the session. Participants were informed about the aims of the study prior to the interviews, and rapport was established before the formal interview. Field notes were taken during and after each interview to capture contextual observations.

### 2.5. Research Team and Reflexivity

This study was conducted by a research team consisting of one male and two female researchers specialized in public health nursing, psychiatric nursing, and obstetrics and gynecological nursing. Two female researchers conducted the interviews. This study was conducted by researchers specializing in public health and psychiatric nursing, as well as obstetrics and gynecological nursing. The interviews were carried out by researchers with expertise in public health and obstetrics and gynecological nursing. The processes of data analysis and coding were conducted collaboratively by all members of the research team. Reflexivity was maintained throughout all stages of the study to prevent the researchers’ professional experiences from influencing the interpretation of the data. To preserve the authenticity of participants’ experiences and to prevent meanings from being overshadowed by researcher interpretations, the analysis process was supported through team-based discussions.

### 2.6. Data Analysis

In this qualitative study, data analysis was conducted based on Colaizzi’s (1978) descriptive phenomenological approach [21]. Initially, statements related to the research question were identified from the participants’ narratives and transformed into conceptual codes. The resulting codes were then grouped under subthemes and overarching themes based on their relationships of meaning. Data obtained from the mothers were analyzed separately prior to the development of a shared phenomenological framework and were subsequently synthesized within a holistic structure. In the presentation of the findings, the COREQ reporting criteria were taken into consideration. Data analysis was conducted manually by the research team following Colaizzi’s phenomenological analysis steps.

Familiarization with the Data: The researchers reviewed the interview transcripts of the mothers through repeated readings to gain immersion in the data and to develop an overall framework.

Identification of Significant Statements: Narratives considered to be directly related to the focus of the study, as well as those that were striking or recurrent, were distinguished and classified as significant statements.

Formulation of Meanings: Each significant statement identified was interpreted in line with its essential meaning, and these interpretations were transformed into conceptual codes.

Theme Development: The codes were grouped under subthemes based on similarities in meaning and content; these subthemes were then integrated into more comprehensive overarching themes. This stage enabled a comparative examination of the findings derived from the mothers.

Exhaustive Description and Identification of the Fundamental Structure: The identified subthemes were integrated to construct a comprehensive description of the phenomenon; subsequently, this description was distilled into a holistic structure reflecting the essential essence of the experience.

Participant Validation: The findings derived from the analysis were shared with the participants, and necessary revisions were made in accordance with their feedback to enhance the validity of the results. Coding and theme development were conducted collaboratively by the research team, and differences in interpretation were resolved through discussion until consensus was reached.

### 2.7. Trustworthiness and Scientific Rigor of the Study

To support the methodological rigor of the study, the following approaches were adopted:

Credibility: The data were analyzed independently by the researchers, and consensus on the shared thematic structure was achieved through comparison of the resulting codes.

Transferability: The demographic characteristics of the participants and the context in which the study was conducted were presented in detail to support the transferability of the findings to similar settings.

Dependability: Methodological consistency in the data collection process was ensured by having all interviews conducted by the same researchers.

Confirmability: Audio recordings, written transcripts, and coding materials were securely stored. The coding and interpretation steps of the analysis process were reviewed by external experts, and revisions were made in accordance with their recommendations.

### 2.8. Researcher Reflexivity

Throughout all stages of the research process, the researchers acted with awareness of their own professional experiences and potential preconceptions. To limit the influence of personal assumptions on data interpretation, a bracketing approach was employed; this process was supported through reflective notes and regular team discussions. During the analysis, researcher influence was continuously monitored in an effort to preserve the objectivity of the findings. To minimize potential researcher bias, reflexive notes were maintained throughout data collection and analysis, and regular team discussions were conducted to bracket prior assumptions.

### 2.9. Ethical Principles

The study was conducted in accordance with the ethical principles outlined in the Declaration of Helsinki. Ethical approval was obtained from the Muş Alparslan University Scientific Research and Publication Ethics Committee (decision No: 15-66; date: 3 December 2025); institutional permission was granted by Muş State Hospital through an official letter dated 2 January 2026 and numbered 35465298. Participants were included in the study on a voluntary basis; they were informed about the purpose of the research, the implementation process, confidentiality principles, and their right to withdraw from the study at any time, and both written and verbal informed consent were obtained.

## 3. Results

The study sample consisted of 20 mothers, with ages ranging from 24 to 42 years. The mean age of the participants was 32.9 years. All participants were married, reflecting a homogeneous marital status profile. In terms of educational attainment, the majority had completed a university degree (*n* = 11), followed by high school education (*n* = 5), primary education (*n* = 2), and postgraduate education (*n* = 2).

Regarding family characteristics, participants had between one and three children. Most mothers had two children (*n* = 9), while others had one child (*n* = 6) or three children (*n* = 5). The age of the youngest child ranged from 3.5 to 12 months, indicating that all participants were within the first postpartum year. The time elapsed since the most recent birth varied accordingly, spanning from 3.5 to 12 months.

With respect to mode of delivery, vaginal birth was more common among participants (*n* = 12), whereas cesarean section was reported by eight mothers. In terms of employment status, nine participants were employed, while eleven were not employed at the time of the study. Detailed demographic characteristics of the participants are presented in Table 1.

### 3.1. Theme 1. Shattering of Existence: The Bifurcation of Identity and the Transition from “I” to “We”

This theme reveals that motherhood constitutes not merely the acquisition of a new social role in the participants’ lived experiences, but rather generates a rupture at an existential level. The postpartum period was experienced by participants as a threshold in which the meaning of life was re-situated, the boundaries of the individual self became blurred, and identity was renegotiated. At this threshold, a life narrative centered on the “I” was suspended; in its place emerged a relational mode of existence shaped around the presence of the baby. Participant narratives indicate that this process unfolds through three intertwined dimensions: the re-situating of life meaning, rupture and questioning in self-perception, and the threshold of transformation—fragile empowerment. The themes and subthemes derived from the analysis are presented in Table 2.

Motherhood profoundly transformed participants’ life purposes and priorities; the relationship established with the body, perceptions of health, and everyday decisions were redefined through the needs of the baby. This re-situating entailed the marginalization of individual needs; life was no longer interpreted through the continuity of a singular subject, but rather through that of a shared “we.” However, this transition was experienced not solely as a natural or spontaneous process of adaptation, but also as a field of tension associated with the dissolution of the individual self.

Participants stated that during the postpartum period, their connection with their former selves weakened, that they sometimes failed to recognize themselves, and that they were confronted with the question “who am I?” This questioning demonstrates that motherhood is not merely a new role added to identity; rather, it is a process that requires the reorganization of the existing structure of the self. While this rupture in self-perception generated feelings of emotional emptiness, alienation, and exhaustion in some participants, for others it also constituted the ground for a potential emergence of new forms of empowerment.

In this context, motherhood was described by participants as neither solely a loss nor entirely a gain. Instead, it was depicted as a transformative space in which vulnerability and empowerment are experienced simultaneously. Increased emotional sensitivity rendered mothers more open and vulnerable; at the same time, it deepened their capacities for endurance, their sense of responsibility, and their existential awareness. This theme reveals motherhood as a multilayered experience that reshapes women’s existential positioning.

*“After becoming a mother, the dreams I had built for myself seem to have been suspended; now I think about life through my child.”*

(P17)

*“Before, if I got sick, I wouldn’t care; now I need not to get sick. The meaning of life has become solely the child.”*

(P1)

*“Now everything in my life is organized around my baby; it is no longer ‘me,’ it is ‘we.’”*

(P14)

*“After becoming a mother, my life split into two—before and after. The time I lived for myself seems to have been left behind.”*

(P11)

This process was also accompanied by a marked rupture in participants’ perceptions of self. Mothers reported that after childbirth, they felt different from their former selves and, at times, even alien to themselves. The question “who am I?” emerged not merely as an identity-related inquiry, but as a form of existential questioning.

*“I am definitely not the same person. These questions exhaust me deeply.”*

(P4)

*“I experienced a disconnection between my old self and my new self; at times I felt as though I fell into a void.”*

(P3)

However, this rupture was experienced not only as loss or dissolution, but also as a transformative space in which a new form of strength began to take root. Participants described motherhood as a state of empowerment that emerges alongside vulnerability, referring to a threshold where emotional sensitivity increases while, at the same time, the capacity to endure expands.

*“I would call it a rebirth, because motherhood gave me an incredible strength.”*

(P1)

*“I am more fragile, but at the same time, stronger.”*

(P3)

### 3.2. Theme 2. Invisible Burdens: Social Vulnerability, Expectations, and Gaps in Support

This theme focuses on the experience of motherhood within a social context and demonstrates that the postpartum period constitutes an intense space of relational vulnerability. Participant narratives reveal that despite the increased caregiving labor associated with motherhood, this labor often remains invisible; the insufficiency of social support mechanisms, combined with societal expectations and a persistent sense of being under surveillance, deepens mothers’ psychosocial burden. This experience is shaped around the dimensions of the collapse of support networks, the pressure of the “good mother” ideal, and social surveillance, alongside loneliness, exclusion, and invisibilization.

Many participants stated that during the postpartum period, they experienced serious deficiencies, particularly in terms of emotional support. The absence of a partner, physical or emotional distance from family members, and the perception of motherhood by the surrounding environment as a “normal” and tolerable process led mothers to shoulder their difficulties alone. This situation resulted in motherhood shifting from a responsibility that should be shared collectively into an individualized struggle.

This vulnerability was further intensified by society’s normative expectations of the “good mother.” Participants reported feeling inadequate, guilty, and unsuccessful due to the constant monitoring, evaluation, and comparison of their mothering practices. This form of social surveillance made it difficult for mothers to trust their own intuitions, transforming the experience of motherhood from an internal, subjective process into a performance that requires external validation.

The persistent sense of being criticized and misunderstood led many mothers to withdraw from their social environments. Participants expressed that the psychological burden they carried was often unrecognized and that the mental and emotional dimensions of motherhood remained invisible. This process of invisibilization deepened feelings of loneliness; mothers came to adopt silence as a coping strategy, relinquishing the pursuit of support.

*“No one saw my burden; everyone acted as if this was simply something I was supposed to do.”*

(P19)

*“I was always alone in my motherhood. There was no support; I was on my own in everything.”*

(P5)

*“My husband was in the military, my family was far away; I was truly alone during this period.”*

(P4)

This sense of loneliness was further deepened by society’s rigid expectations of the “good mother.” Mothers reported experiencing intense feelings of inadequacy and guilt due to the constant monitoring, criticism, and comparison of their mothering practices.

*“The anxiety of being a perfect mother brought me to the point of regretting becoming a mother.”*

(P1)

*“I was judged for giving formula, for expressing milk; I felt inadequate.”*

(P10)

These pressures led mothers, over time, to withdraw from their social environments and to experience an increasing sense of invisibility. Participants stated that the difficulties they faced were often trivialized and that the psychological burden of motherhood was largely unrecognized.

*“You struggle, but everyone says ‘we all went through it’; it is as if a mother’s mental health does not matter.”*

(P16)

*“As I felt increasingly misunderstood, I distanced myself from people.”*

(P14)

### 3.3. Theme 3. Re-Tailoring the Self: Identity Reconstruction and Inner Transformation

This theme demonstrates that motherhood initiates a long-term and dynamic process of reconstruction within participants’ sense of self. Rather than being a fixed identity that is automatically established at birth, the maternal identity was experienced by participants as one that is shaped over time through experience, adaptation, and internal negotiation. This process unfolded along the axes of integration with the maternal identity, the distinction between the old self and the new self, and role conflict and identity balance.

Participants reported that motherhood was initially perceived as a heavy, unfamiliar, and demanding role; however, over time, this role became internalized. For some participants, the maternal identity coexisted with acceptance and adaptation, while for others, it was accompanied by ambivalence and longing. This situation indicates that motherhood is not a singular or homogeneous experience, but rather a multidimensional process shaped by individual histories, expectations, and life circumstances.

With the transition to motherhood, marked changes occurred in participants’ self-perceptions. Priorities, emotional responses, and personal boundaries were redefined; mothers described themselves as more patient and more responsible, yet simultaneously more anxious and sensitive individuals. This differentiation between the old self and the new self required mothers to reorganize the relationship they established with themselves.

In this process, role conflict emerged as a significant area of tension. The attempt to simultaneously sustain the roles of mother, partner, worker, and individual generated feelings of imbalance and exhaustion among participants. Many mothers reported that the maternal role came to overshadow their other identities and that this situation at times strained their sense of self. In this context, the self ceased to be a fixed structure and, together with the experience of motherhood, assumed a form that was continuously “re-tailored.”

*“There is a great difference between who I was before becoming a mother and who I am now; it feels as though I am reshaping myself.”*

(P8)

*“Motherhood turned me into a completely different person; I am still trying to get to know myself again.”*

(P16)

*“At first it felt very heavy, but now the maternal identity is a part of me.”*

(P3)

*“I couldn’t accept it at first, but over time I came to love being a mother.”*

(P7)

Throughout this process, mothers perceived clear differences between their former selves and their new maternal selves. Priorities, emotional responses, and personal boundaries shifted; mothers described themselves as more patient, yet simultaneously more anxious individuals.

*“It feels as though another person emerged from within me; my priorities changed completely.”*

(P9)

*“I am more patient, but also much more anxious.”*

(P2)

Role conflict constituted one of the most challenging dimensions of this transformation. The maternal role often took precedence over other identities, and mothers reported difficulty in maintaining balance.

*“It is very difficult to carry all of them at once; I couldn’t establish balance.”*

(P2)

*“Most of the time, I neglected my partner and myself.”*

(P3)

### 3.4. Theme 4. The Silent Construction of Resilience: Inner Strategies, Spiritual Anchoring, and Hope

This theme reveals the processes of inner resilience that mothers developed over time despite their intense experiences of vulnerability, loneliness, and role burden. Participant narratives indicate that resilience was not experienced as a sudden or dramatic state of empowerment; rather, it emerged as a quiet, gradual, and often invisible process woven into everyday life. This process was shaped around spirituality and inner resources, practices of self-repair, and orientation toward the future and the reconstruction of hope.

Spirituality emerged as one of the primary ways through which many participants generated meaning in the face of uncertainty and loss of control. Motherhood was conceptualized by some mothers as a sacred responsibility, a trust, or an existential duty. This mode of meaning-making enabled the reframing of experienced difficulties along the axes of destiny, patience, and responsibility, thereby creating an inner anchoring space that supported psychological resilience.

Participants developed individual strategies aimed at self-repair over time. Crying, withdrawing inward, lowering expectations, setting boundaries, and consciously disregarding certain societal judgments emerged as key components of this process. These practices were shaped not through professional support, but rather through personal awareness and lived experience, functioning as protective mechanisms that enabled mothers to cope with emotional burden.

Hope, as reflected in participant narratives, emerged not as an abstract expectation oriented toward a distant future, but as a practical form of coping continuously reproduced within everyday life. Mothers made sense of the future not through long-term ideals, but through “getting through today,” “completing one more day,” and “holding on to small gains.” In this respect, hope functioned not as a force that eliminated vulnerability, but rather as a fundamental psychological building block that could coexist with it and sustain mothers’ connection to life.

*“Sometimes it feels as though nothing will ever get better, yet I keep going; this gives me strength.”*

(P15)

*“Telling myself every day, ‘I got through today as well,’ keeps me standing.”*

(P20)

*“There is a trust that has been given to me; I have to act accordingly.”*

(P12)

*“Motherhood made me feel complete.”*

(P18)

Over time, mothers developed individual strategies aimed at self-repair. Crying, withdrawing inward, setting boundaries, or consciously ignoring certain expectations were among these practices.

*“In time, I learned to listen to myself and to stop caring.”*

(P4)

*“Sometimes crying was my only way of coping.”*

(P3)

For participants, hope evolved into a form of anchoring that was reconstructed within everyday life rather than remaining an abstract expectation. Mothers made sense of the future through small steps rather than grand goals.

*“I have to be strong for my children.”*

(P14)

*“It is a difficult, yet hopeful journey.”*

(P7)

## 4. Discussion

This phenomenological study reveals how women reconstruct their identities and frameworks of meaning during the postpartum period and how this process is interwoven with social vulnerabilities and profound existential transitions. The findings indicate that motherhood is not merely a biological transformation but a multilayered psychosocial process involving the dissolution and reconstruction of the self, as well as the re-creation of life’s meaning. The themes identified are consistent with approaches in the literature that conceptualize the transition to motherhood as an ontological transformation [22,23].

The present study demonstrates that the transition to motherhood is experienced not merely as the addition of a new role to an existing identity, but as an existential rupture that disrupts the continuity of the self. Women’s shift from an “I”-centered perception to a “mother–child”-oriented mode of existence necessitates the repositioning and reconfiguration of identity. Priyadharshini and Karthiga (2025) reported that the early motherhood period profoundly disrupts women’s self-perception and that the maternal identity takes precedence over previous social and professional identities [24]. Similarly, Odunsi and Hosek (2024) emphasize that labels attributed to motherhood prompt women to renegotiate their core identities [25]. It is noteworthy that this rupture demonstrates cross-cultural continuity. Migrant mothers associate motherhood with a loss of personal space and existential questioning [26]. It has also been reported that working mothers experience uncertainty and a need for readjustment [27]. The intensity of cultural and ethical expectations may lead women to postpone their individual aspirations and develop a more relational sense of self [28,29]. In this context, the transition to motherhood can be regarded not merely as a change in roles but as an ontological threshold at which the subject’s relationship with the world is fundamentally reconfigured.

The study demonstrates that a significant proportion of women encounter invisible social burdens during the postpartum period. The ideal of the “perfect mother” positions motherhood as a natural and effortless process, thereby reducing the visibility of the difficulties women experience. Benuyenah and Tran (2020) demonstrated that societal expectations increase unexpressed distress and feelings of loneliness among single mothers [30]. It has also been reported that first-time mothers experience increased vulnerability when they lack adequate social support [31]. Participants’ tendency to limit help-seeking behaviors due to concerns about being perceived as weak points to the regulatory influence of social norms on psychological help-seeking processes. The literature consistently demonstrates that low levels of social support increase the risk of postpartum stress and depression [32]. It has been reported that among migrant mothers, language and cultural barriers complicate the process of seeking support [33,34] and that structural inequalities are determinant factors influencing postpartum mental health [35]. Accordingly, social vulnerability may be considered a phenomenon more closely related to the societal organization of care than to individual inadequacy.

The study indicates that the initial disruption in identity gradually gives way to a process of reconstruction over time. Women’s internalization of motherhood and their re-organization of priorities support the notion that identity is not fixed but rather a structure that transforms through lived experience. Delgado Pérez et al. (2021) state that postpartum transformation can contribute to women’s rediscovery of themselves and to the strengthening of their self-esteem [3]. Identity reconstruction is an ongoing process of negotiation between the former and the emerging selves rather than a linear process of adjustment. This transition has been described as a continuous negotiation [27], and previous studies have demonstrated that identity transformation is shaped through interaction with cultural norms and individual history [25,36]. Although the search for balance among maternal, spousal, and professional roles may lead to role conflict, it has been reported that women are able to develop adaptive strategies over time [37,38]. This process suggests that identity is not only reconstructed but also reinterpreted by mothers within a newly established framework of meaning.

One of the significant findings of the study is that vulnerability and resilience are not mutually exclusive but can coexist simultaneously. Participants reported enhancing their coping capacities through strategies such as daily rituals, emotional boundaries, and cognitive reframing. Findings demonstrating the association between spiritual well-being and a lower risk of depression [39] support the notion that spirituality may function as a psychological protective resource. It has also been reported that spiritual practices facilitate the attribution of meaning to hardships [10,40]. Resilience appears to be reinforced through hope and micro-level coping strategies. It has been reported that Iranian mothers reconstruct their sense of hope through daily goals [41] and that Chinese mothers develop cognitive and relational adaptation in response to cultural pressures [42]. Additionally, psychological resilience has been shown to play a mitigating role in reducing symptoms of postpartum depression and post-traumatic stress [43]. These findings suggest that meaning-making may serve as a central mechanism in adaptation to motherhood.

This study demonstrates that the reconstruction of identity and meaning in the postpartum period is a multilayered, relational, and culturally embedded process. Motherhood may initially create an existential disruption that interrupts the continuity of the self, and social expectations may further deepen this vulnerability. Nevertheless, over time, women are able to mobilize their internal and relational resources to reconstruct their identities and develop a more integrated framework of meaning.

From a theoretical perspective, these findings suggest that the transition to motherhood cannot be explained solely through role adaptation; rather, ontological transformation, meaning-making, and relational identity perspectives should be considered together [22,23]. At the clinical and societal levels, it is essential that postpartum care not focus solely on physical recovery but be structured according to holistic models that encompass identity transformation, social support, and spiritual needs. Such an approach may not only reduce mothers’ vulnerabilities but also support their adaptation processes.

### Limitations

This study has several limitations. First, the research was conducted in a single province in eastern Türkiye, and the sociocultural characteristics of the region may have influenced mothers’ experiences; therefore, the transferability of the findings to other contexts should be considered with caution. Second, the study employed a qualitative phenomenological design with a purposive sample, which prioritizes depth of understanding rather than statistical generalization. Finally, although the study explored social vulnerability through participants’ narratives and contextual characteristics, future research using longitudinal or mixed-method designs may provide a more comprehensive understanding of identity reconstruction and meaning-making during the postpartum transition.

## 5. Conclusions

This phenomenological study demonstrates that the postpartum period is not merely a phase in which women assume the maternal role, but rather an existential transitional space in which identity, selfhood, and the meaning of life are reconstructed. The findings reveal that the transition to motherhood generates an ontological repositioning from an “I”-centered existence toward a relational “we” orientation, corresponding to a profound inner transformation in which women metaphorically give birth to themselves. This rebirth unfolds within a paradoxical structure in which vulnerability and empowerment are experienced simultaneously.

The study demonstrates that identity reconstruction is not independent of the social context; elements such as invisible caregiving labor, the ideal of the “good mother,” and limited social support transform motherhood into an individualized struggle, thereby increasing the psychosocial burden. Conversely, the internal coping practices women develop, their spiritual orientations, and the hope reconstituted in everyday life suggest that the postpartum period represents not only a rupture but also a ground for meaning-making and the development of resilience.

Motherhood appears to constitute a dynamic configuration that evolves over time rather than a fixed identity; as women strive to establish a balance between their former selves and their maternal identities, they develop a deeper existential awareness. In this respect, the postpartum period represents a critical threshold at which dissolution and integration intersect.

From a nursing practice perspective, the findings indicate that postpartum care should extend beyond physical recovery and be addressed through a holistic approach encompassing women’s identity-related and existential needs. Psychosocial care models that recognize and support this process of “giving birth to oneself” may strengthen healthy identity integration.

In conclusion, the postpartum period represents a profound transformative experience in which a woman not only gives birth to a baby but also reconstructs her own existence. Making this process visible, developing sensitive professional approaches to understand mothers’ vulnerabilities, and supporting their transitional experiences should be among the primary priorities of women’s health services.

### Recommendations

Brief phenomenological interviews assessing women’s sense of identity continuity, self-perception, and search for meaning should be integrated into postpartum follow-up protocols. This approach may render visible not only clinical risks but also early signals of the dissolution and reconstruction of the self.

Considering the “invisible labor” and experiences of isolation indicated by the findings, structured home visits within the first year and continuity-based nurse-led contact models should be developed. Continuity supports the establishment of a trust-based relationship in which mothers can share their experiences free from performative expectations.

Motherhood education programs should be redesigned with realistic content that emphasizes the normative nature of identity disruption and ambivalence, rather than reproducing the idealized discourse of the “good mother.” Such a framework may help women make sense of their internal tensions without pathologizing their experiences.

In care plans, it should be acknowledged that not only the infant but also the mother is a “newly emerging subject,” and reflective counseling practices that support women’s adaptation to this metaphorical process of giving birth to themselves should be incorporated.

In in-service training programs for healthcare professionals, reflective clinical skills that foster awareness of the existential dimension of the transition to motherhood—such as deep listening, meaning-oriented questioning, and tolerance of silence—should be strengthened.

Future research is recommended to employ longitudinal qualitative designs that address postpartum identity reconstruction as a temporal process and to examine its relationship with social support structures in a multilevel manner.

## Figures and Tables

**Table 1:** Sociodemographic and obstetric characteristics of the participants (*n* = 20).

Participant	Age	Education Level	Marital Status	Number of Children	Age of Youngest Child	Mode of Delivery	Time Since Last Birth	Employment Status
P1	30	University degree	Married	1	9 months	Vaginal delivery	9 months	Employed
P2	35	University degree	Married	2	7 months	Cesarean section	7 months	Employed
P3	34	University degree	Married	2	7 months	Cesarean section	7 months	Employed
P4	30	High school	Married	1	12 months	Vaginal delivery	12 months	Unemployed
P5	38	Primary education	Married	3	12 months	Vaginal delivery	12 months	Unemployed
P6	28	University degree	Married	1	9 months	Cesarean section	9 months	Employed
P7	24	High school	Married	2	8.5 months	Vaginal delivery	8.5 months	Unemployed
P8	34	University degree	Married	2	9.5 months	Vaginal delivery	9.5 months	Unemployed
P9	35	University degree	Married	2	5.5 months	Cesarean section	5.5 months	Employed
P10	30	University degree	Married	2	12 months	Vaginal delivery	12 months	Unemployed
P11	25	High school	Married	1	6 months	Vaginal delivery	6 months	Unemployed
P12	34	University degree	Married	3	3.5 months	Vaginal delivery	3.5 months	Unemployed
P13	35	University degree	Married	3	11 months	Vaginal delivery	11 months	Employed
P14	42	University degree	Married	2	11 months	Cesarean section	11 months	Unemployed
P15	26	Primary education	Married	2	7 months	Vaginal delivery	7 months	Employed
P16	29	High school	Married	2	12 months	Cesarean section	12 months	Employed
P17	34	University degree	Married	2	5 months	Vaginal delivery	5 months	Unemployed
P18	30	High school	Married	1	4 months	Vaginal delivery	4 months	Unemployed
P19	36	Postgraduate degree	Married	2	4 months	Cesarean section	4 months	Employed
P20	29	Postgraduate degree	Married	1	12 months	Vaginal delivery	12 months	Unemployed

**Table 2:** Main themes, subthemes, and representative participant quotations derived from the phenomenological analysis.

Main Theme	Subthemes	Brief Description	Representative Participant Quotations
Theme 1. Shattering of Existence: The Bifurcation of Identity and the Transition from “I” to “We”	1.1. Re-Situating the Meaning of Life	The shift of life’s center from the mother to the baby in the postpartum period; the transformation of meaning from an individual “I” to a relational “we.”	“Before, if I got sick, I wouldn’t care; now it matters a lot, I need not to get sick… The meaning of life has become solely child-centered.” (P01)\“After my baby was born, my priorities changed; now ‘we’ are at the center of life.” (P02)
	1.2. Rupture and Questioning in Self-Perception	A split between the old and the new self; an identity rupture characterized by the question “who am I?”	“It feels as if my self is divided in two; half me, half my baby.” (P01)\“I experienced a disconnection between my old self and my new self.” (P03)
	1.3. Threshold of Transformation: Fragile Empowerment	The experience of motherhood as a transformation that is simultaneously exhausting and empowering.	“I would call it a rebirth; motherhood empowered me.” (P01)\“After childbirth, I felt as if I was complete.” (P14)
Theme 2. Invisible Burdens: Social Vulnerability, Expectations, and Gaps in Support	2.1. Collapse of Support Networks	Insufficient support from family and the social environment; the experience of motherhood as an individualized burden.	“I could not receive the necessary support from my family; I felt very helpless.” (P01)\“Because I am a healthcare professional, they said ‘you can handle it.’” (P02)
	2.2. The Pressure of the “Good Mother” Ideal and Social Surveillance	Feelings of guilt, inadequacy, and regret generated by societal expectations.	“The anxiety of being a perfect mother made me regret becoming a mother.” (P01)\“I behaved like someone I am not in order to meet societal expectations.” (P02)
	2.3. Loneliness, Exclusion, and Invisibility	Increasing loneliness and social withdrawal accompanied by a sense of being misunderstood.	“I felt extremely lonely, excluded, and misunderstood.” (P01)\“I usually found relief by crying.” (P02)
Theme 3. Re-Tailoring the Self: Identity Reconstruction and Inner Transformation	3.1. Integration with the Maternal Identity	The gradual acceptance and internalization of the maternal identity.	“It was difficult at first, but now the maternal identity is a part of me.” (P10)\“Over time, I have come to embrace this identity.” (P01)
	3.2. Distinction Between the Old Self and the New Self	Marked changes in personality traits, priorities, and social relationships.	“Almost everything changed; it feels as though another person emerged from within me.” (P02)\“My current self is more anxious.” (P01)
	3.3. Role Conflict and Identity Balance	Difficulties in balancing the roles of mother, partner, worker, and individual.	“Trying to manage all of them together is quite difficult.” (P01)\“I want to work, but my maternal role predominates.” (P07)
Theme 4. The Silent Construction of Resilience: Inner Strategies, Spiritual Anchoring, and Hope	4.1. Spirituality and Inner Resources	The function of spirituality as a source of meaning and strength in the face of uncertainty.	“Motherhood gave me an incredible strength.” (P01)\“During this process, I felt complete.” (P14)
	4.2. Self-Repair Practices	The development of patience, emotional regulation, and inner coping strategies.	“I learned to be patient.” (P02)\“Over time, I became more resilient.” (P03)
	4.3. Holding on to the Future and the Reconstruction of Hope	A conception of hope focused on sustaining daily life rather than pursuing grand goals.	“Every day, I feel that I am becoming a little more patient.” (P02)\“This process gave me a new strength.” (P10)

## Data Availability

The data presented in this study are available on request from the corresponding author.

## Abbreviations

The following abbreviations are used in this manuscript:

COREQ	Consolidated Criteria for Reporting Qualitative Research
FHCs	Family Health Centers
